# Identification of Circulating MicroRNAs as a Promising Diagnostic Biomarker for Cervical Intraepithelial Neoplasia and Early Cancer: A Meta-Analysis

**DOI:** 10.1155/2020/4947381

**Published:** 2020-03-23

**Authors:** Yao Jiang, Zuohong Hu, Zhihua Zuo, Yiqin Li, Fei Pu, Biqiong Wang, Yan Tang, Yongcan Guo, Hualin Tao

**Affiliations:** ^1^Department of Clinical Laboratory Medicine, The Affiliated Hospital of Southwest Medical University, Luzhou, China; ^2^Department of Pathology, Chengdu First People's Hospital, Chengdu, China; ^3^Department of Oncology, The Affiliated Hospital of Southwest Medical University, Luzhou, China; ^4^Department of Clinical Laboratory Medicine, Jinniu Maternity And Child Health Hospital of Chengdu, Chengdu, China; ^5^Clinical Laboratory of Traditional Chinese Medicine Hospital Affiliated to Southwest Medical University, Luzhou, China

## Abstract

**Background:**

Cervical cancer (CC) is one of the most common female malignant tumors. And cervical intraepithelial neoplasia (CIN) is the precancerous lesion of CC, which can progress to invasive CC. MicroRNAs (miRNAs) have been found to be potential diagnostic biomarkers for CIN or CC. However, recently, the lack of sufficient studies about the diagnostic value of miRNAs for CIN made it challenging to separately investigate the diagnostic efficacy of miRNAs for CIN. Likewise, the conclusions among those studies were discordant. Therefore, we conducted this meta-analysis, aimed at evaluating the diagnostic efficacy of miRNAs for CIN and CC patients.

**Methods:**

Literature search was performed in PubMed, Embase, and Web of Science databases. Pooled sensitivity, specificity, and other diagnostic parameters were calculated through Stata 14.0 software. Furthermore, subgroup analyses and metaregression analysis were conducted to explore the main sources of heterogeneity.

**Results:**

Ten articles covering 50 studies were eligible, which included 5,908 patients and 4,819 healthy individuals. The pooled sensitivity, specificity, positive likelihood ratio (PLR), negative likelihood ratio (NLR), diagnostic odds ratio (DOR), and area under the curve (AUC) were 0.81 (95% CI, 0.77-0.85), 0.86 (95% CI, 0.83-0.89), 5.9 (95% CI, 4.5-7.7), 0.22 (95% CI, 0.17-0.28), 27 (95% CI, 17-44), and 0.91 (95% CI, 0.88-0.93), respectively. Additionally, the ethnicity and internal reference were the main sources of heterogeneity.

**Conclusions:**

Circulating miRNAs can be a promising noninvasive diagnostic biomarker for CIN and early CC, especially miR-9 and miR-205, which need to be verified by large-scale studies.

## 1. Introduction

Cervical cancer (CC) remains the second leading cause of female malignant tumors and the third most common cause of cancer-related deaths among females in underdeveloped countries [1, 2]. The incidence of invasive CC was 8.9 per 100,000 women between 1998 and 2003 [3]. A total of 100,700 new cases were diagnosed as CC in China, which accounted for 6.16% of female malignant tumors in 2013 [4]. From 2015 to 2030, the estimated mortality rate of CC will increase by approximately 22% in the whole world [5]. And the American Cancer Society (ACS) has estimated that 13,170 new cases will be diagnosed as CC and 4,250 women will die from this cancer in 2019 [6]. Before progressing to the CC, the cervical intraepithelial neoplasia (CIN) is the precancerous lesion of CC, including mild atypical hyperplasia (CIN1), moderate atypical hyperplasia (CIN2), severe atypical hyperplasia, and carcinoma in situ (CIS) (together called CIN3). Generally, CIN1 is regarded as low-grade lesion due to the regression of most lesions and conservative treatment. By contrast, CIN2/CIN3, which are considered as high-grade lesions, have been proved to be associated with cell transformation induced by human papillomavirus (HPV) oncogenes, with the potential of progression to the invasive tumor [7]. Therefore, identification of CIN or early CC is extremely significant. So far, a large number of methods for screening CIN or early CC have been developed, such as HPV DNA testing [8], papanicolaou (pap) smear [9], liquid-based cytology (LBC) [10], joint test, and colposcopy, leading to the reduction of the incidence and mortality rate of CC [11–13]. Nevertheless, existing screening methods were complained of some limitations, including false-positive rate [14] or false-negative rate [15–17], possibility of overdiagnosis [18], probability of missed diagnosis [19], invasive procedure (cervical scraping or tissue biopsy), the difference between interobserver and intraobserver, and variation among pathologists [20], which made the triage of screening CC more complicated [21]. Therefore, it is extremely imperative to find simple, noninvasive, and feasible biomarkers for identification of CIN and early CC.

MicroRNAs (miRNAs), evolutionarily conserved small noncoding RNA with 21-23 nucleotides, play a key role in regulating gene expression [22] through complete or incomplete pairing with mRNA 3′UTR, which results in mRNA degradation or inhibits mRNA translation into protein, respectively [23]. MiRNAs participate in many biological processes, such as proliferation, differentiation, apoptosis [24], hematopoietic differentiation, and the expression of oncogene or tumor suppressor genes [25]. At present, miRNAs have been proved to be diagnostic biomarkers of multiple tumors, including gastric cancer [26], pancreatic cancer [27], non-small cell lung cancer [28], bladder cancer [29], cervical cancer [30], and CIN [19]. However, insufficient studies about the diagnostic value of miRNAs for CIN made it difficult to separately investigate the diagnostic efficacy of miRNAs for CIN. Additionally, the existence of inconsistent conclusions among those studies encouraged us to explore the possibility of miRNAs as diagnostic biomarkers of CIN and CC patients. For example, Zheng et al. found that exosomal let-7d-3p and miR-30d-5p were capable of differentiating CIN II+ group from CIN I- group (including CINI patients and healthy subjects), with area under the curve (AUC) of being 0.828 [30]. By contrast, Zhang et al. indicated that four circulating miRNAs (miR-16-2^∗^, miR-195, miR-2861, and miR-497) had moderate diagnostic efficacy in discriminating CIN patients from healthy individuals (AUC = 0.734) [31]. Besides, Ma et al. utilized three datasets (training set, testing set, and validation set) to evaluate the diagnostic value of miRNA panel (miR-146a-5p, miR-151a-3p, miR-2110, and miR-21-5p) for CC, with AUC of being 0.911, 0.774, and 0.786, respectively [32]. Due to the significantly inconsistent conclusions about the capability of miRNAs for diagnosis of CIN or CC, it is necessary to investigate the diagnostic efficacy of miRNAs for CIN or CC patients through a meta-analysis. In addition, the diagnostic meta-analyses of miRNAs for CIN or CC also were scarce. Similarly, most of meta-analyses focused on the diagnostic value of the HPV DNA testing [33–35], cytology [36–38], or combination of HPV DNA testing and cytology [39, 40] for CIN or CC patients. Therefore, we conducted this meta-analysis, aimed at evaluating the diagnostic efficacy of miRNAs for CIN and CC patients, which might provide some useful information for clinician about early identification of CIN or CC.

## 2. Material and Methods

### 2.1. Search Strategy

We searched the key terms in PubMed, Embase, and Web of Science databases using relevant search formula without restriction of the language and publication date, with the deadline of November 14, 2019. And the medical subject headings (MeSH) and entry words were obtained on the National Center for Biotechnology Information (NCBI) website. The search terms were as follows: (“uterine cervical neoplasms” OR “cervical neoplasms” OR “cancer of the uterine cervix” OR “cervical cancer” OR “cervix neoplasms” OR “cervical intraepithelial neoplasia” OR “cervical intraepithelial neoplasms”) AND (“miRNAs” OR “microRNAs” OR “miR^∗^”) AND (“diagnos^∗^”). In addition, the relevant articles also were manually searched.

### 2.2. The Criteria of Inclusion and Exclusion

The process of screening was independently performed by two reviewers (Yao Jiang and Zuohong Hu). The inclusion criteria were as follows: (1) studies about the diagnostic value of miRNAs in distinguishing CIN and CC patients from healthy individuals; (2) the data must be complete for calculating the value of true positives (TP), false positives (FP), false negatives (FN), and true negatives (TN). By contrast, the studies would be excluded if they were reviews, meta-analyses, conferences, duplicates, irrelevant to the diagnosis of CIN and CC, or the studies with insufficient data. When encountering the disagreements, we solved these problems by discussion.

### 2.3. Data Extraction and Quality Assessment

The two reviewers separately extracted the data of included studies, which contained the first author, sample size, publication year, country, ethnicity, mean age of participants, sample type, detection methods of miRNAs, internal reference, miRNA profiling, cut-off values, sensitivity, specificity, and AUC with 95% confidence intervals (CIs). Then, the quality of studies was evaluated by the quality assessment of diagnostic accuracy studies (QUADAS-2) tool [41] using RevMan 5.3 software. The quality assessment scale contained patient selection, index test, reference standard, and flow and timing domains, which had 2-3 questions in every domain for assessing the risk of bias. And all of the domains except the flow and timing domain also needed to evaluate the applicability concerns.

### 2.4. Statistical Analysis

For evaluating the diagnostic value of miRNAs for CIN and CC, we extracted the sample size, sensitivity, and specificity in every study, where these data can be used to calculate the value of TP, FP, FN, and TN through RevMan 5.3 software. The statistical analysis of meta-analysis was conducted using Stata 14.0 software, including the pooled sensitivity, specificity, positive likelihood ratio (PLR), negative likelihood ratio (NLR), diagnostic odds ratio (DOR), summary receiver operating characteristic curves (sROC) with 95% CIs. Additionally, the value of AUC in sROC curve is 0.5-0.7, 0.7-0.9, and 0.9-1.0, which represents the low, moderate, and high diagnostic efficacy, respectively [42]. In addition, we also explored the threshold effect based on Spearman's correlation coefficient and *P* value through Meta-DiSc 1.4 software [43]. And the heterogeneity between studies was evaluated by *Q* test and the *I*^2^ statistics. The value of *I*^2^ was more than 50%, and *P* value was less than 0.05, indicating the significant heterogeneity [44], then, a random-effect model was selected [45]. Subsequently, the main sources of heterogeneity [44] were investigated via subgroup analyses and metaregression analysis. Furthermore, the Deek's funnel plot was used to assess the potential publication bias. The *P* value was less than 0.1, showing that there existed the publication bias [46].

## 3. Results

### 3.1. Characteristics of Included Studies

**Figure 1:**
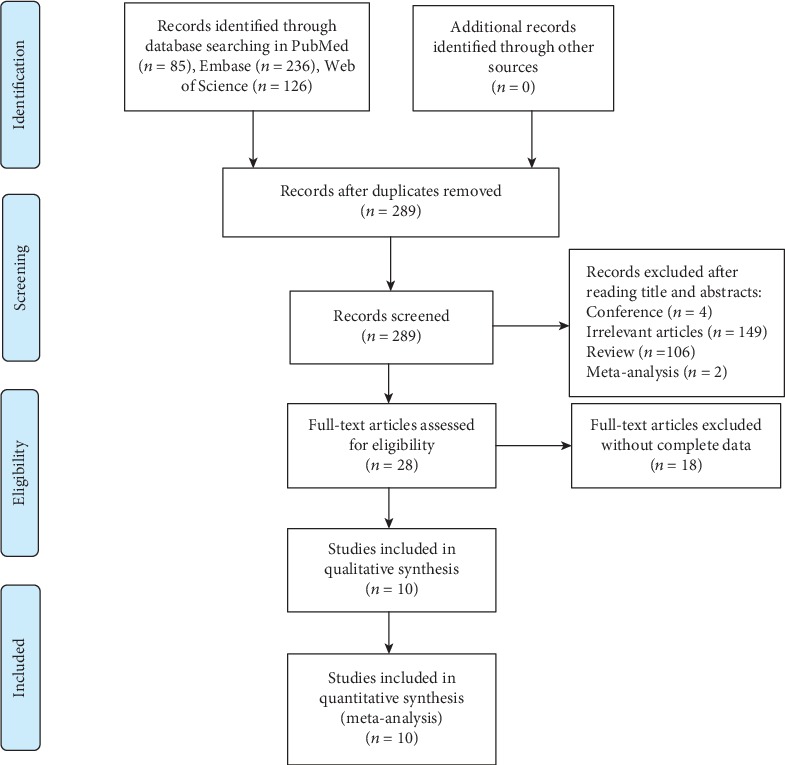
The flowchart of screening the eligible studies.

In total, we found 447 articles in three databases. After removing 158 duplicate articles, we included the eligible studies through reading titles and abstracts. Ultimately, ten articles [31, 47–55], covering 50 studies, were included according to the inclusion and exclusion criteria.  Figure 1 showed the flow diagram of study selection. All of studies were published in English, and the publication year ranged from 2015 to 2019. There were 5,908 patients with CIN or CC and 4,819 healthy participants.  Table 1 showed the main characteristics of included studies.

### 3.2. Quality Assessment

The overall quality of studies was barely satisfactory. Most of studies adopted case-control study design, and all of patients were diagnosed as CIN or CC by pathologists. Although Babion et al. [55] avoided the case-control design, they did not mention the appropriate exclusion criteria, which caused the unclear bias risk in patient selection domain. With respect to the flow and timing domain, Jia et al. [48], Liu et al. [50], and Nagamitsu et al. [53] did not include all patients in the diagnostically two by two contingency table. [Supplementary-material supplementary-material-1] showed the detailed results of quality assessment scale in four domains.

### 3.3. Diagnostic Value of miRNAs

**Figure 2:**
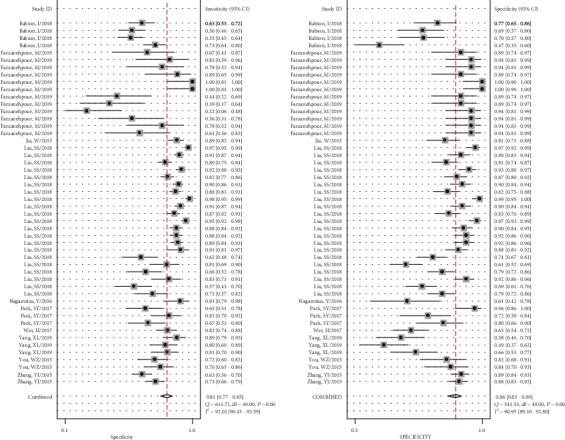
Forest plots show the sensitivity and specificity of miRNAs in the diagnosis of CIN and CC, respectively. The dots represent the effect size of a single study. And the diamond represents the pooled effect size of included studies.

**Figure 3:**
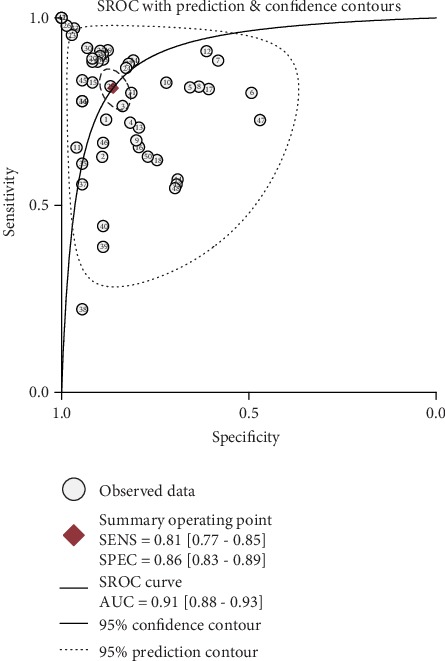
Summary receiver operating characteristic (sROC) curve of miRNAs in diagnosis of CIN and CC.

**Figure 4:**
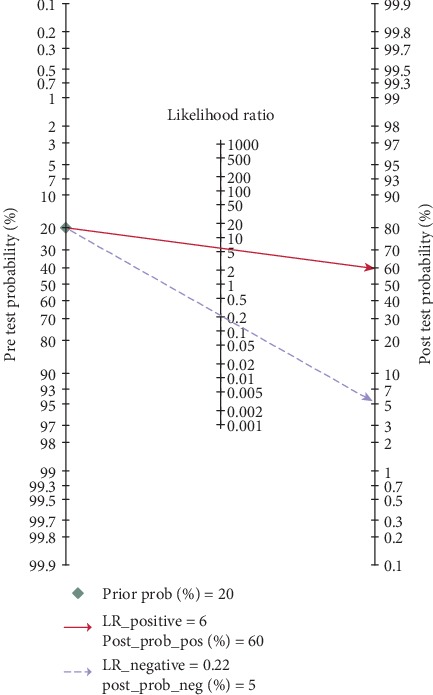
Fagan plot evaluates the clinical utility of miRNAs for distinguishing CIN and CC patients from healthy individuals. The pretest probability was set to 20%. The red solid line represents 60% posttest probability of being CIN or CC when miRNAs were dysregulated. The blue dotted line shows that the posttest probability of participants being diagnosed as CIN or CC was 5% in case miRNAs were at normal level.

As shown in Figure 2, on account of the *I*^2^ more than 50% (92.02% for sensitivity and 90.95% for specificity) in the forest plot, a random-effect model was selected to evaluate the diagnostic efficacy of miRNAs for CIN and CC patients. The pooled results were as follows: sensitivity, 0.81 (95% CI, 0.77-0.85); specificity, 0.86 (95% CI, 0.83-0.89); PLR, 5.9 (95% CI, 4.5-7.7); NLR, 0.22 (95% CI, 0.17-0.28); DOR, 27 (95% CI, 17-44); and AUC, 0.91 (95% CI, 0.88-0.93) (Figure 3), indicating that miRNAs can be potential biomarkers in differentiating CIN and CC patients from healthy participants. Next, threshold effect was investigated using Meta-DiSc 1.4 software, with Spearman's correlation coefficient of -0.304 and a *P* value of 0.032, showing the existence of threshold effect. A PLR of 5.9 suggested that the abnormal expression of miRNAs had 5.9 times possibility of accurately identifying CIN and CC patients from healthy individuals. And the results of NLR (0.22) indicated when miRNAs were at normal expression level, the expected probability of being diagnosed as CIN or CC was 22%. Additionally, a Fagan plot was shown in Figure 4. In case the prior probability was set to 20%, the positive posttest and negative posttest possibilities were 60% and 5%, respectively. In other words, if miRNAs were dysregulated, the participants had possibility of being CIN or CC was 60%. On the contrary, individuals had a 5% chance of being diagnosed as CIN or CC when the miRNAs were normal expression.

### 3.4. Subgroup Analyses and Metaregression Analysis

**Figure 5:**
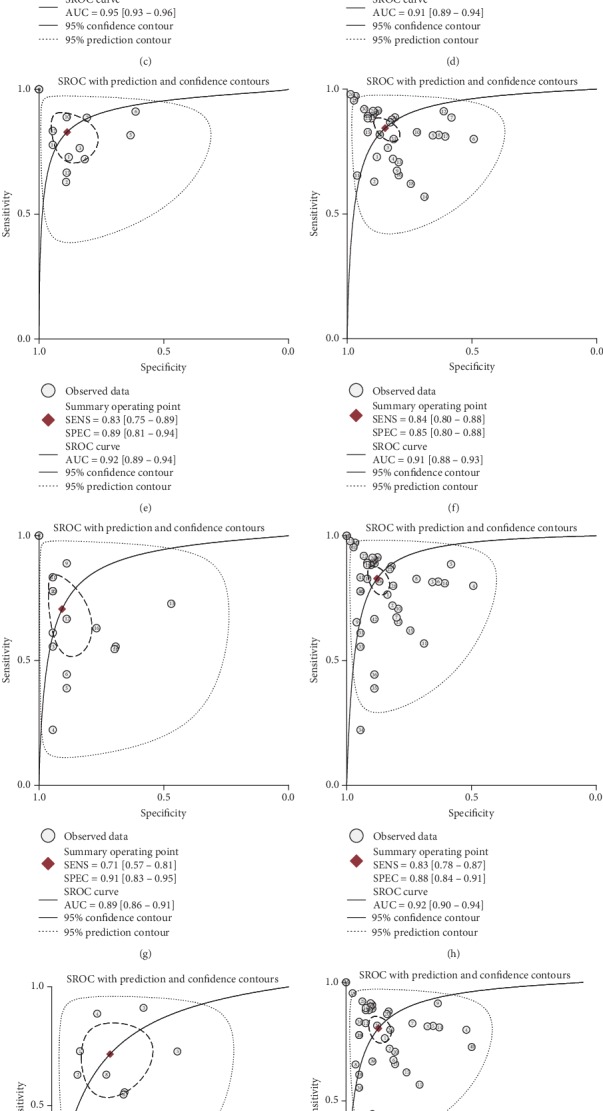
Summary receiver operating characteristic curves show the diagnostic efficacy of miRNAs in (a) CIN *vs* healthy controls, (b) CC *vs* healthy controls, (c) serum miRNAs, (d) tissue miRNAs, (e) circulating miRNAs, (f) Asian, (g) Caucasian, (h) U6 as internal reference, (i) other miRNAs as internal reference, (j) single miRNA, and (k) miRNA panel subgroups, respectively.

Considering the heterogeneity was considerable in our meta-analysis with *I*^2^ more than 50%, subgroup analyses and metaregression analysis were performed. Intriguingly, the diagnostic efficacy of miRNAs in discriminating CIN patients from healthy subjects was remarkable (sensitivity: 0.82, 95% CI: 0.75-0.88; specificity: 0.89, 95% CI: 0.84-0.92; PLR: 7.2, 95% CI: 5.0-10.4; NLR: 0.20, 95% CI: 0.13-0.30; DOR: 36, 95% CI: 18-75; and AUC: 0.93, 95% CI: 0.90-0.95) (Figure 5(a)). Besides, when acting as diagnostic biomarkers in distinguishing CC patients from healthy individuals, miRNAs had moderate diagnostic efficacy, with the sensitivity, specificity, PLR, NLR, DOR, and AUC of 0.79 (95% CI, 0.74-0.83), 0.83 (95% CI, 0.76-0.88), 4.6 (95% CI, 3.3-6.5), 0.25 (95% CI, 0.20-0.32), 18 (95% CI, 11-30), and 0.87 (95% CI, 0.84-0.90), respectively (Figure 5(b)). In addition, we found that serum-derived miRNAs had the highest discriminatory power for CIN and CC patients (sensitivity: 0.86, 95% CI: 0.75-0.93; specificity: 0.92, 95% CI: 0.83-0.96; PLR: 10.2, 95% CI: 4.8-21.7; NLR: 0.15, 95% CI: 0.08-0.30; DOR: 66, 95% CI: 19-225; and AUC: 0.95, 95% CI: 0.93-0.96) (Figure 5(c)). Nevertheless, most of the studies (*n* = 33) adopted the cervical tissues to assess the diagnostic value of miRNAs (sensitivity: 0.82, 95% CI: 0.76-0.87; specificity: 0.87, 95% CI: 0.83-0.90; PLR: 6.3, 95% CI: 4.6-8.6; NLR: 0.21, 95% CI: 0.15-0.28; DOR: 30, 95% CI: 17-53; and AUC: 0.91, 95% CI: 0.89-0.94) (Figure 5(d)), inferior to serum-derived miRNAs. Since the cervical tissues were harvested using invasive methods, it is not convenient to routinely screen the population in clinic. Therefore, we investigated the diagnostic efficacy of circulating miRNAs for CIN or CC patients, including serum and plasma miRNAs, with sensitivity, specificity, PLR, NLR, DOR, and AUC of being 0.83 (95% CI, 0.75-0.89), 0.89 (95% CI, 0.81-0.94), 7.4 (95% CI, 4.1-13.2), 0.19 (95% CI, 0.13-0.30), 38 (95% CI, 16-93), and 0.92 (95% CI, 0.89-0.94), respectively (Figure 5(e)), showing that circulating miRNAs can be promising diagnostic biomarkers of CIN and CC. In addition, the ethnicity also had impact on the diagnostic value of miRNAs, with the Asian (AUC: 0.91, 95% CI: 0.88-0.93) (Figure 5(f)) higher than Caucasian (AUC: 0.89, 95% CI: 0.86-0.91) (Figure 5(g)). Moreover, U6 was usually selected as internal reference in the majority of studies, with the sensitivity, specificity, PLR, NLR, DOR, and AUC of being 0.83, 0.88, 6.8, 0.19, 35, and 0.92, respectively (Figure 5(h)), which was more diagnostically accurate than choosing other miRNAs as internal reference, such as cel-miR-67 [31], miR-423-3p [55], miR-16 [53], and let-7 [48] (Figure 5(i)). Surprisingly, single miRNA (Figure 5(j)) and miRNA panel (Figure 5(k)) had a similar diagnostic efficacy. Meanwhile, we found some specific miRNAs, including miR-9 ([Supplementary-material supplementary-material-1]) and miR-205 ([Supplementary-material supplementary-material-1]), which can be candidate molecular markers for identification of CIN and CC patients.  Table 2 showed all the results of subgroup analyses. Due to the quality assessment having higher or unclear bias risks, the quality of studies was not taken into consideration for subgroup analyses and metaregression analysis.

**Figure 6:**
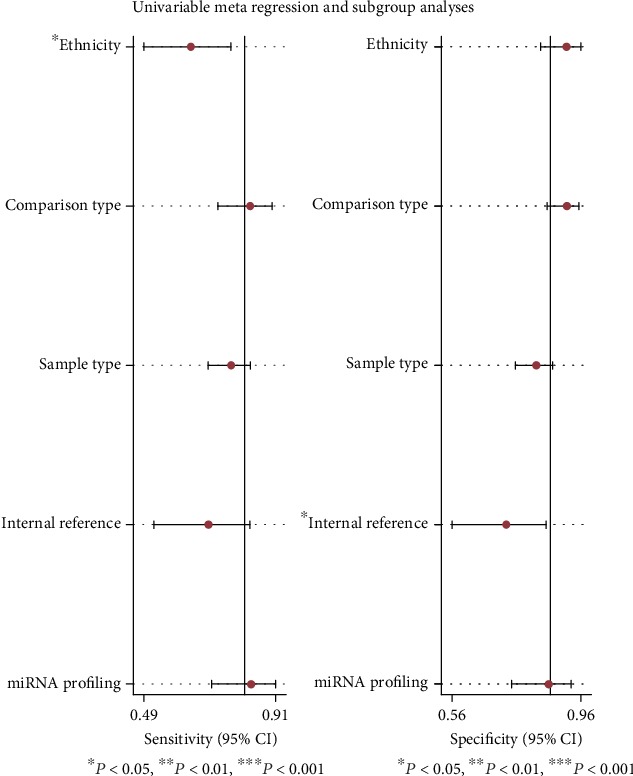
Univariable metaregression and subgroup analyses for exploring the main sources of heterogeneity. ^∗^ in ethnicity and internal reference showed that the *P* value was less than 0.05, and the difference was statistically significant.

As for metaregression analysis, we found that the ethnicity and internal reference were the main sources of heterogeneity, with the *P* value less than 0.05 (Figure 6).

### 3.5. Publication Bias

**Figure 7:**
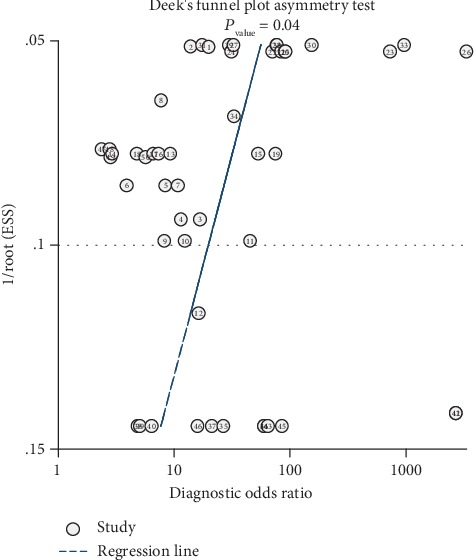
Deek's funnel plot evaluates the potential publication bias.

Publication bias was evaluated by the Deek's funnel plot. As shown in Figure 7, the *P* value was 0.04, far less than 0.1, indicating that the publication bias did exist.

## 4. Discussions

CC is one of the most common gynecological malignant tumors [56], which is mainly attributed to persistent infection with sexually transmitted high-risk HPV types [57, 58]. The symptoms and signs of early CC are usually not obvious, making the diagnosis challenging, especially early identification of CIN in healthy population. Recently, miRNAs have been proved to be diagnostic biomarkers in many tumors [59, 60], which might provide deep insight into the diagnostic value of miRNAs for CIN and CC patients [61]. However, due to the inconsistent conclusions among those studies, as well as insufficient studies about the diagnostic value of miRNAs for CIN, we conducted this meta-analysis, aimed at evaluating the diagnostic efficacy of miRNAs for CIN and CC patients.

Our results showed that miRNAs can be a promising biomarker for participating in the diagnosis of CIN and CC before performing the HPV DNA testing or Pap smears, with higher diagnostic efficacy (AUC: 0.91, 95% CI: 0.88-0.93). Additionally, we found that miRNAs were capable of discriminating CIN individuals from healthy controls with remarkable diagnostic performance. CIN, the precancerous lesions of CC, is a category of continuous disease associated with morphological changes [7] in cervical squamous cells [62]. In case CIN individuals are timely detected and intervened, they will not progress to invasive CC, which might decrease the incidence and mortality rate of CC [63]. Additionally, the DOR, positively correlated with AUC, is an indicator of discriminatory test performance, ranging from 0 to infinity. And the higher of DOR value is, the better of diagnostic efficacy will be [64]. MiRNAs had the ability to discriminate CIN individuals from healthy participants with a DOR of being 36, suggesting that miRNAs can be promising molecular markers for early identification of CIN individuals, which might promote the development of the diagnostic biomarkers of CIN and early CC in clinic. Considering the miRNAs are stable in circulating system, we investigated whether the circulating miRNAs and serum-derived miRNAs might be diagnostic biomarkers of CIN and CC. Intriguingly, circulating miRNAs, especially serum-derived miRNAs, had more outstanding diagnostic efficacy than cervical tissues, which needed invasive manipulation to harvest samples. Likewise, Jia et al. found that serum miRNA panel as the diagnostic fingerprint of CC had sensitivity, specificity, and AUC of 88.6%, 81%, and 0.908, respectively [48]. In addition, Farzanehpour et al. showed that serum miR-192 can be a potential diagnostic biomarker for early detection of CC, with 83.3% sensitivity, 94.4% specificity, and 0.98 AUC [54]. Luo et al. demonstrated that serum miR-3142 was significantly upregulated in CC patients, with the AUC of being 0.935 (95% CI: 0.893-0.977) [65]. Additionally, Juan et al. identified two novel serum miRNAs by Solexa sequencing, finding that the two serum miRNAs can be biomarkers associated with the diagnosis of CC [66]. Therefore, circulating miRNAs, especially serum-derived miRNAs, can be optimal noninvasive biological markers for diagnosis of CIN or early CC. Subsequently, we explored the diagnostic efficacy of some specific miRNAs, including miR-9 and miR-205, which had favorable diagnostic efficacy. According to the findings of Zhang et al., inhibition of miR-9 could induce apoptosis of CC cells by combining to FOXO3 gene, providing potential molecular targets for CC patients [67]. Likewise, Aishanjiang et al. also found that miR-9 was overexpressed in CC lines and clinical tissues, which can directly target FOXO1 gene to enhance invasion and migration of CC [68]. Moreover, Farzanehpour et al. showed that miR-9 had AUC of 0.99 in distinguishing CC patients from healthy individuals, with 100% sensitivity and 94.4% specificity [54]. For miR-205, Xie et al. found that elevated miR-205 expression had significantly higher specificity than the high-risk HPV DNA testing, and its sensitivity was similar to the high-risk HPV DNA testing, which can predict CIN2 and CIN3 squamous intraepithelial lesions in women with low-grade squamous intraepithelial lesions (LSIL), but not high-risk women [69]. And You et al. also indicated that miR-205 can discriminate CC patients from healthy subjects, with 72.0% sensitivity, 82.35% specificity, and 0.843 AUC [51]. Thus, miR-9 and miR-205 can act as the promising noninvasive diagnostic biomarkers in identifying CIN or early CC patients.

Our meta-analysis had some advantages compared with previous studies. First of all, miRNAs had better diagnostic efficacy in discriminating the CIN and CC patients from healthy individuals, which broaden our horizons about the diagnostic biomarkers of CIN or CC. Secondly, the remarkable diagnostic efficacy of miRNAs in differentiating CIN individuals from healthy participants provided new insight into miRNAs for diagnosis of CIN patients, which can promote the development of diagnostic biomarkers of CIN or early CC, especially CIN individuals. Ultimately, we explored the diagnostic value of some specific miRNAs for CIN and early CC, including miR-9 and miR-205, which might provide useful information for clinician in early diagnosis of CIN or CC patients using miR-9 or miR-205 in the future.

However, the limitations also cannot be ignored. The presence of publication bias might be associated with those studies with small sample size, lack of studies with negative results, and one article covering multiple studies. Additionally, due to the case-control study design, the risk bias of quality assessment was high or unclear. Meanwhile, threshold effect did exist in our meta-analysis, which might be caused by the different cut-off values. For instance, Park et al. set three different cut-off values in detection of the expression level of miR-9, miR-21, and miR-155, with the values of 4.035, 1.975, and 3.88, respectively [49]. According to the metaregression analysis, we found that the ethnicity and internal reference were the main sources of heterogeneity. Therefore, a large quantity of multiple-central studies and unified internal reference are needed to reduce the occurrence of heterogeneity in the future. Although these findings were promising, large sample size studies and a mass of prospective high-quality studies are needed to verify our findings.

## 5. Conclusions

Therefore, circulating miRNAs, especially miR-9 and miR-205, can be promising noninvasive diagnostic biomarkers for CIN and early CC patients, which need to be verified by large-scale studies.

## Figures and Tables

**Table 1:** The characteristics of included studies.

Author	Year	Country	Ethnicity	Comparison type	Sample size		Mean age(year)		Sample type	Method	Reference	miRNAs profiling
					Case	Control	Case	Control				
Babion, I	2018	Netherlands	Caucasian	CIN3 vs HC	121	66	35	41	Exfoliated cells	qRT-PCR	miR-423-3p	miR-125b-5p, miRNA panel
Babion, I	2018	Netherlands	Caucasian	CIN3 vs HC	108	65	NA	NA	Exfoliated cells	qRT-PCR	miR-423-3p	miRNA panel
Farzanehpour, M	2019	Iran	Caucasian	CC vs HC	18	36	61	36	Tissues	qRT-PCR	U6 snRNA	miR-9, miR-192, miR-205
Farzanehpour, M	2019	Iran	Caucasian	CIN vs HC	18	36	47	36	Tissues	qRT-PCR	U6 snRNA	miR-9, miR-192, miR-205
Farzanehpour, M	2019	Iran	Caucasian	CC vs HC	18	36	61	36	Serum	qRT-PCR	U6 snRNA	miR-9, miR-192, miR-205
Farzanehpour, M	2019	Iran	Caucasian	CIN vs HC	18	36	47	36	Serum	qRT-PCR	U6 snRNA	miR-9, miR-192, miR-205
Jia, W	2015	China	Asian	CC vs HC	123	94	46	47.8	Serum	RT-qPCR	Mixture of let-7i, -7 g and -7d	5 miRNAs
Liu, SS	2018	China	Asian	CC vs HC	58	145	51	50	Tissues	RT-qPCR	RNU6B	miR-20a, miR-92a, miR-141, miR-183^∗^, miR-210, miR-944, miRNA panel
Liu, SS	2018	China	Asian	LG-CIN vs HC	239	145	39	50	Tissues	RT-qPCR	RNU6B	miR-20a, miR-92a, miR-141, miR-183^∗^, miR-210, miR-944, miRNA panel
Liu, SS	2018	China	Asian	HG-CIN vs HC	285	145	42	50	Tissues	RT-qPCR	RNU6B	miR-20a, miR-92a, miR-141, miR-183^∗^, miR-210, miR-944, miRNA panel
Nagamitsu, Y	2016	Japan	Asian	CC vs HC	45	31	49	39	Serum	RT-qPCR	miR-16	miR-1290
Park, SY	2017	Korea	Asian	CC vs HC	52	50	NA	NA	Tissues	RT-qPCR	RNU6B	miR-9, miR-21, miR-155
Wei, H	2017	China	Asian	CC vs HC	120	120	NA	NA	Plasma	RT-qPCR	RNU6B	miR-145
Yang, XL	2019	China	Asian	CC vs HC	70	67	40.2	41.1	Tissues	RT-qPCR	U6	miR-1202, miR-195, miRNA panel
You, WZ	2015	China	Asian	CC vs HC	68	49	45	42	Plasma	RT-qPCR	RNU6B	miR-127, miR-205
Zhang, YJ	2015	China	Asian	CC vs HC	184	193	NA	NA	Serum	qPCR	Cel-miR-67	miRNA panel
Zhang, YJ	2015	China	Asian	CIN vs HC	186	193	NA	NA	Serum	qPCR	Cel-miR-67	miRNA panel

CC: cervical cancer; HC: healthy controls; CIN: cervical intraepithelial neoplasia; NA: not available.

**Table 2:** The results of subgroup analyses in meta-analysis.

Subgroups	No. of studies	SEN (95% CI)	SPE (95% CI)	PLR (95% CI)	NLR (95% CI)	DOR (95% CI)	AUC (95% CI)
*Comparison type*							
CC vs HC	25	0.79 (0.74, 0.83)	0.83 (0.76, 0.88)	4.6 (3.3, 6.5)	0.25 (0.20, 0.32)	18 (11, 30)	0.87 (0.84, 0.90)
CIN vs HC	25	0.82 (0.75, 0.88)	0.89 (0.84, 0.92)	7.2 (5.0, 10.4)	0.20 (0.13, 0.30)	36 (18, 75)	0.93 (0.90, 0.95)
*Sample type*							
Serum	10	0.86 (0.75, 0.93)	0.92 (0.83, 0.96)	10.2 (4.8, 21.7)	0.15 (0.08, 0.30)	66 (19, 225)	0.95 (0.93, 0.96)
Tissues	33	0.82 (0.76, 0.87)	0.87 (0.83, 0.90)	6.3 (4.6, 8.6)	0.21 (0.15, 0.28)	30 (17, 53)	0.91 (0.89, 0.94)
Circulating miRNAs	13	0.83 (0.75, 0.89)	0.89 (0.81, 0,94)	7.4 (4.1, 13.2)	0.19 (0.13, 0.30)	38 (16, 93)	0.92 (0.89, 0.94)
*Ethnicity*							
Asian	34	0.84 (0.80, 0.88)	0.85 (0.80, 0.88)	5.5 (4.2, 7.4)	0.18 (0.14, 0.24)	30 (18, 50)	0.91 (0.88, 0.93)
Caucasian	16	0.71 (0.57, 0.81)	0.91 (0.83, 0.95)	7.6 (3.7, 15.6)	0.32 (0.21, 0.50)	24 (8, 69)	0.89 (0.86, 0.91)
*Internal reference*							
U6	42	0.83 (0.78, 0.87)	0.88 (0.84, 0.91)	6.8 (5.1, 9.2)	0.19 (0.15, 0.26)	35 (21, 59)	0.92 (0.90, 0.94)
Others	8	0.72 (0.61, 0.80)	0.75 (0.65, 0.84)	2.9 (2.0, 4.3)	0.38 (0.26, 0.54)	8 (4, 15)	0.80 (0.76, 0.83)
*miRNAs profiling*							
Single miRNA	40	0.81 (0.76, 0.85)	0.86 (0.82, 0.90)	5.9 (4.4, 7.9)	0.22 (0.18, 0.28)	26 (17, 42)	0.90 (0.87, 0.93)
miRNA panel	10	0.83 (0.69, 0.92)	0.86 (0.75, 0.92)	5.9 (3.0, 11.8)	0.19 (0.09, 0.41)	30 (8, 120)	0.91 (0.89, 0.94)
miR-9	5	0.73 (0.37, 0.93)	0.94 (0.84, 0.98)	13.2 (3.4, 50.8)	0.28 (0.09, 0.90)	47 (5, 476)	0.95 (0.93, 0.97)
miR-205	5	0.66 (0.53, 0.77)	0.88 (0.82, 0.92)	5.7 (3.8, 8.5)	0.38 (0.27, 0.54)	15 (8, 28)	0.89 (0.86, 0.92)

CC: cervical cancer; HC: healthy controls; CIN: cervical intraepithelial neoplasia; SEN: sensitivity; SPE: specificity; PLR: positive likelihood ratio; NLR: negative likelihood ratio; DOR: diagnostic odds ratio; AUC: area under the curve.

## Data Availability

The data used to support the findings of this study are included within the article.

## Supplementary Materials

Supplementary 1 Figure S1: a summary table of quality assessment.

Supplementary 2 Figure S2: SROC curve for assessing the diagnostic value of miR-9 in differentiating CIN and CC patients from healthy participants.

Supplementary 3 Figure S3: SROC curve for evaluating the diagnostic efficacy of miR-205 in distinguishing CIN and CC patients from healthy individuals.
