# Supplementary figures and images for: Identification of Circulating MicroRNAs as a Promising Diagnostic Biomarker for Cervical Intraepithelial Neoplasia and Early Cancer: A Meta-Analysis
Source: Biomed Res Int. 2020 Mar 23;2020:4947381. doi: 10.1155/2020/4947381 (PMC7125453; doi:10.1155/2020/4947381)

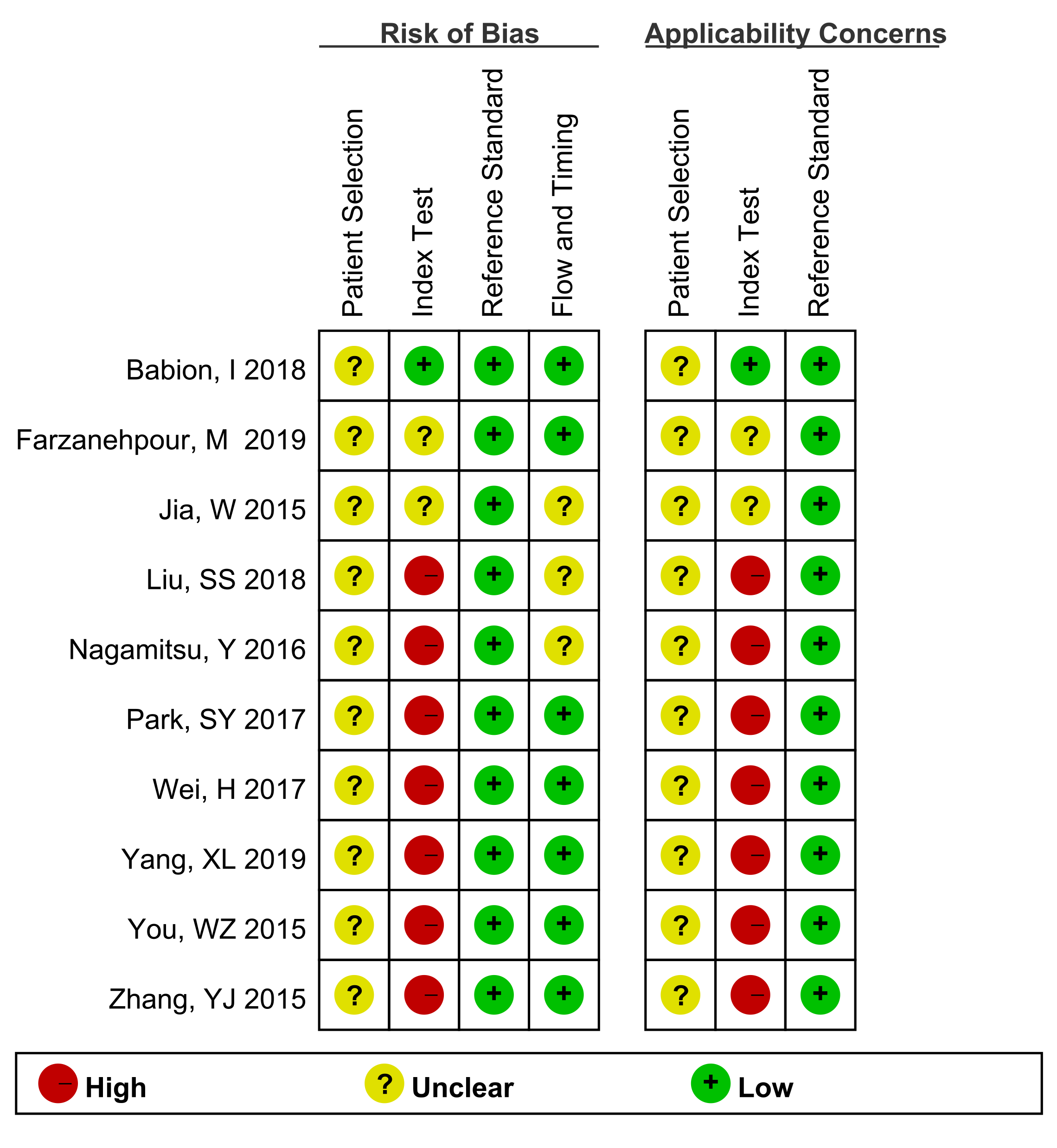

Supplement: Supplementary 1 — Figure S1: a summary table of quality assessment. [file 4947381.f1.tif]

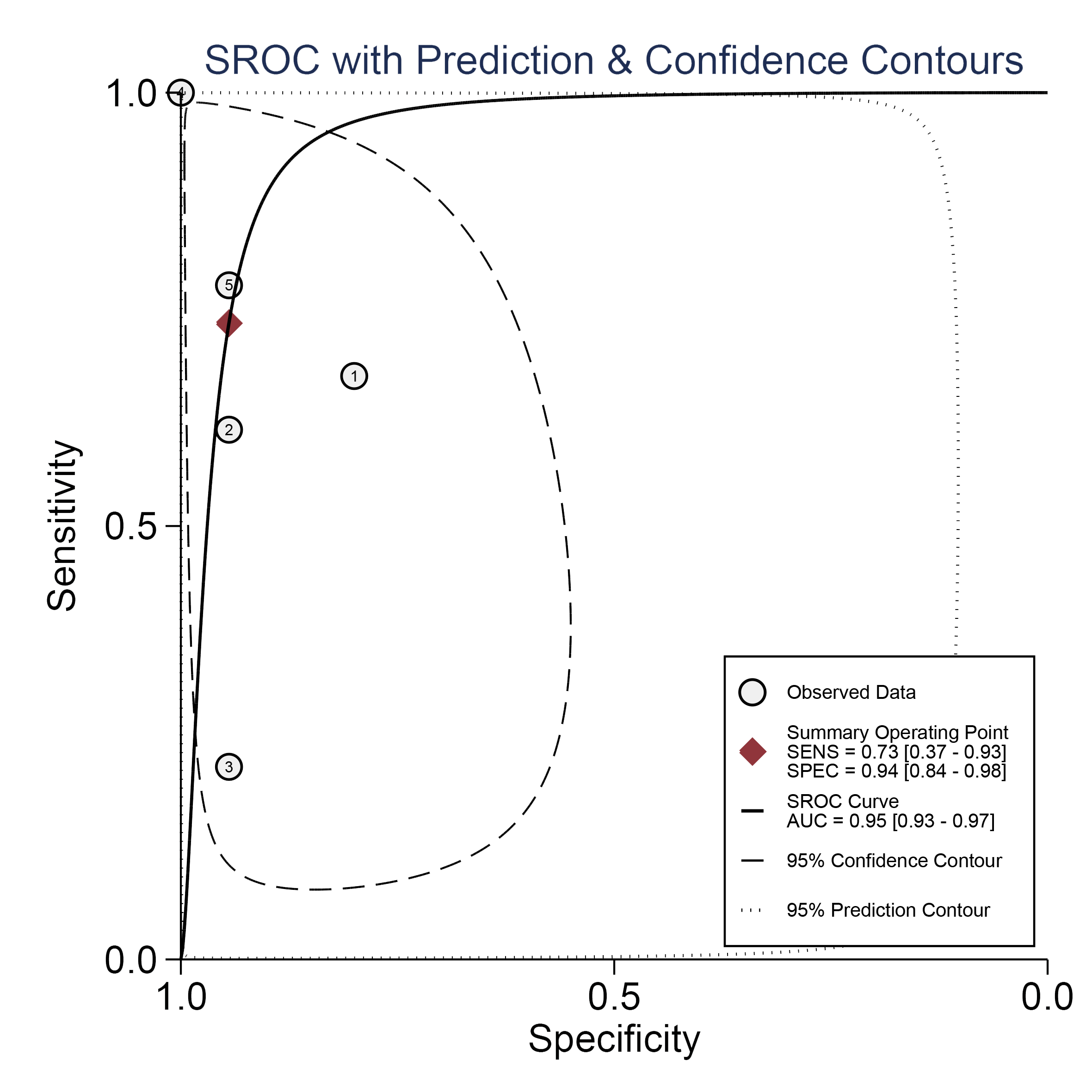

Supplement: Supplementary 2 — Figure S2: SROC curve for assessing the diagnostic value of miR-9 in differentiating CIN and CC patients from healthy participants. [file 4947381.f2.tif]

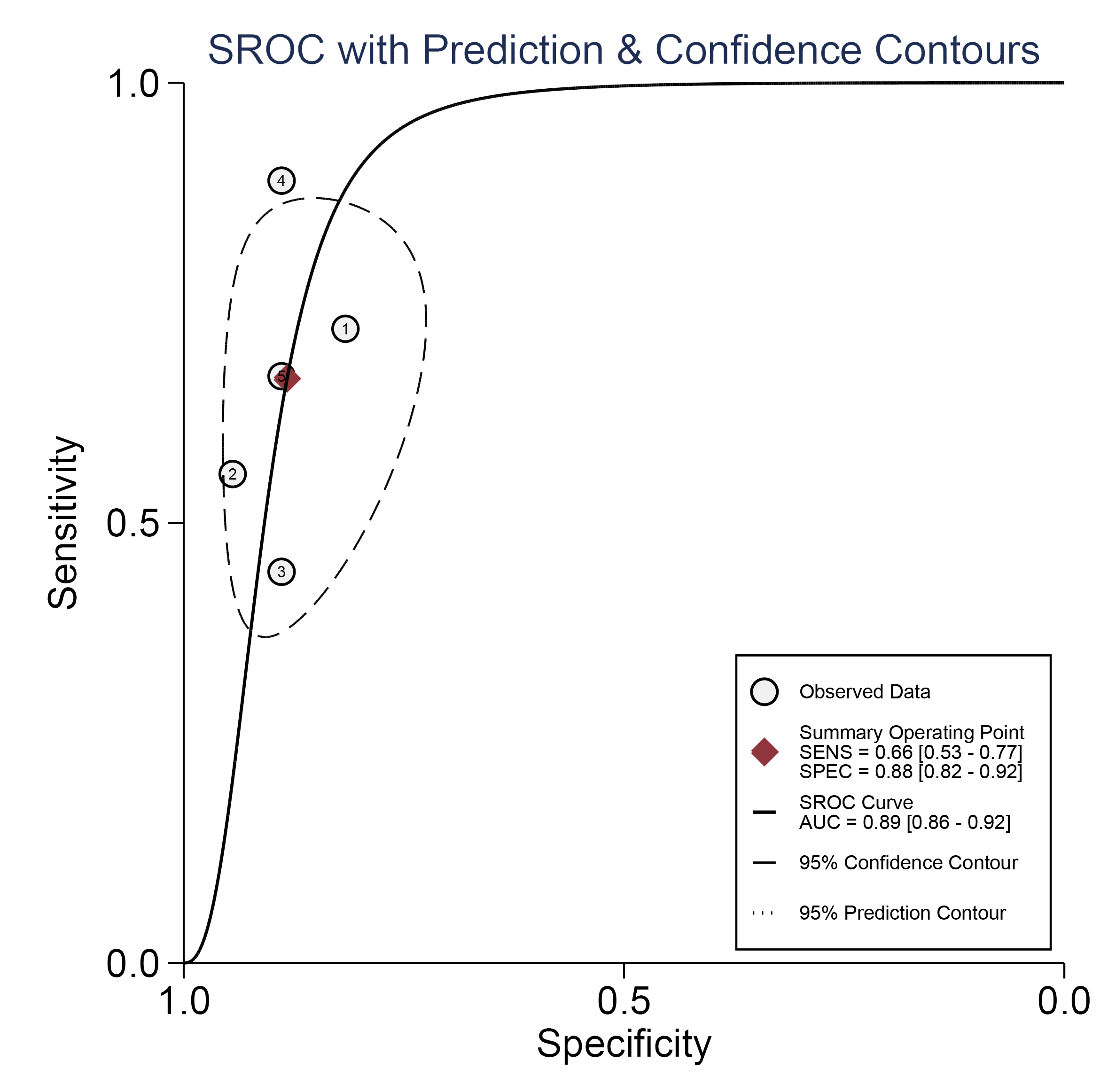

Supplement: Supplementary 3 — Figure S3: SROC curve for evaluating the diagnostic efficacy of miR-205 in distinguishing CIN and CC patients from healthy individuals. [file 4947381.f3.tif]
